# Altered gut microbiota and short chain fatty acids in Chinese children with autism spectrum disorder

**DOI:** 10.1038/s41598-018-36430-z

**Published:** 2019-01-22

**Authors:** Simeng Liu, Enyao Li, Zhenyu Sun, Dongjun Fu, Guiqin Duan, Miaomiao Jiang, Yong Yu, Lu Mei, Pingchang Yang, Youcai Tang, Pengyuan Zheng

**Affiliations:** 1 Department of Gastroenterology, the Fifth Affiliated Hospital of Zhengzhou University, Zhengzhou, 450052 China; 2 Department of Children Rehabilitation Medicine, the Fifth Affiliated Hospital of Zhengzhou University, Zhengzhou, 450052 China; 3 School of Pharmaceutical Sciences, Zhengzhou University, Zhengzhou, Henan 450001 China; 4 Center of Children Psychology and Behavior, the Third Affiliated Hospital of Zhengzhou University, Zhengzhou, 450052 China; 5 Brain Body Institute, McMaster University, Hamilton, ON Canada; 6 Department of Pediatrics, the Fifth Affiliated Hospital of Zhengzhou University, Zhengzhou, 450052 China

## Abstract

Autism spectrum disorder (ASD) is a neurodevelopmental disorder that is characterized by impairments in social interactions and communication, restricted interests and repetitive behaviors. Several studies report a high prevalence of gastrointestinal (GI) symptoms in autistic individuals. Cumulative evidence reveals that the gut microbiota and its metabolites (especially short-chain fatty acids, SCFAs) play an important role in GI disorders and the pathogenesis of ASD. However, the composition of the gut microbiota and its association with fecal SCFAs and GI symptoms of autistic children remain largely unknown. In the present study, we sequenced the bacterial 16S rRNA gene, detected fecal SCFAs, assessed GI symptoms and analyzed the relationship between the gut microbiome and fecal SCFAs in autistic and neurotypical individuals. The results showed that the compositions of the gut microbiota and SCFAs were altered in ASD individuals. We found lower levels of fecal acetic acid and butyrate and a higher level of fecal valeric acid in ASD subjects. We identified decreased abundances of key butyrate-producing taxa (*Ruminococcaceae, Eubacterium, Lachnospiraceae and Erysipelotrichaceae*) and an increased abundance of valeric acid associated bacteria (*Acidobacteria*) among autistic individuals. Constipation was the only GI disorder in ASD children in the present study. We also found enriched *Fusobacterium*, *Barnesiella, Coprobacter* and valeric acid-associated bacteria (*Actinomycetaceae*) and reduced butyrate-producing taxa in constipated autistic subjects. It is suggested that the gut microbiota contributes to fecal SCFAs and constipation in autism. Modulating the gut microbiota, especially butyrate-producing bacteria, could be a promising strategy in the search for alternatives for the treatment of autism spectrum disorder.

## Introduction

Autism spectrum disorder is characterized by persistent deficits in social interactions and communication combined with restricted, repetitive patterns of behaviors, interests, and activities^1^. The estimated male to female ratio of ASD is 4–5:1, and the reported prevalence of ASD is 14.6/1,000 in children at eight years of age^2^. ASD imposes a heavy healthy and economic burden on autistic individuals, their families and society. It is well accepted that both genetic and environmental factors contribute to the etiology of ASD^3,4^.

Emerging evidence indicates that the composition of the gut microbiota and microbial metabolites are altered in a wide range of diseases, including ASD^5^. The human gut harbors trillions of microorganisms and contains approximately 10^14^ bacterial genes that belong to approximately 1,000 species^6^. The gut microbiota communicates with the central nervous system (CNS) through endocrine, immune, metabolic and neural pathways^7^. Communication occurs via a complex bidirectional system known as the “microbiome-gut-brain axis”, which plays an important role in physiology, brain development, immune and metabolic homeostasis^8^. Disturbance of the microbiome-gut-brain axis may be associated with the pathogenesis of ASD.

Short-chain fatty acids (SCFAs) are the functional and vital components in the microbiome-gut-brain axis. SCFAs mainly consist of acetic acid (AA), propionic acid (PPA), butyrate (BTA) and valeric acid (VA). It is reported that PPA disturbs GI function in a manner similar to the abnormalities in ASD^9^. Butyrate inhibits histone deacetylases, suppresses intestinal pro-inflammatory macrophage function^10^, and regulates the blood-brain barrier (BBB) and gut permeability^11^, which are altered in ASD^12^. However, the published data on fecal SCFAs in autism are inconsistent.

Cumulative evidence demonstrates that gastrointestinal (GI) disorders are common comorbidities in autistic children. GI symptoms, such as constipation, abdominal pain, diarrhea and flatulence, have a close relationship with ASD^13^. Furthermore, Adams suggested that GI problems are associated with the severity of autism^14^. Campbell revealed genetic evidence supporting the association between GI disorders and ASD^15^. However, it has not been determined which GI symptoms are associated with autistic children.

Additionally, the composition of the gut microbiota and its association with fecal SCFAs and GI symptoms in autistic children remain largely unknown. In this study, we sequenced the V3-V4 hypervariable regions of the bacterial 16S ribosomal RNA gene, detected fecal SCFAs and assessed the GI symptoms of autistic and neurotypical children to better understand the role of the gut microbiota in ASD.

## Results

### The ASD group has a higher incidence of constipation than the NT group

The mean age of autistic and neurotypical children was 4.43 ± 1.47 and 4.28 ± 1.00 years, respectively (*P* = 0.177). The sex ratio (boys to girls) of autistic subjects was 25:5 (indicating a male gender bias in autism) and was 16:4 for neurotypical children. There were eight children (26.67%) with a moderate level of constipation and one child (3.33%) with a severe level of constipation out of 30 autistic subjects. Constipation was significantly higher in autistic subjects than that in neurotypical subjects (only 1 out of 20 normal subjects had a moderate level of constipation) (*P* < 0.05). None of the patients suffered from diarrhea or abdominal pain. We found no significant differences between autistic and neurotypical subjects in regard to the average stool consistency, stool smell and flatulence. In addition, there was no significant difference in the total GI scores between the two groups (Table 1).

**Table 1:** Characteristics and GI symptom scores of participants.

	Autistic	Neurotypical	*p* value
Subjects (n)	30	20	−
Age (years)	4.43 ± 1.47	4.28 ± 1.00	n.s.
Gender			n.s.
Male	25 (83%)	16 (80%)	
Female	5 (17%)	4 (20%)	
Constipation			0.035*
0	21 (7%)	19 (95%)	
1	8 (26.7%)	1 (5%)	
2	1 (3.3%)	0 (0%)	
Diarrhea			1.000
0	30 (100%)	20 (100%)	
1	0 (0%)	0 (0%)	
2	0 (0%)	0 (0%)	
Average stool consistency		0.771	
0	29 (96.7%)	19 (95%)	
1	1 (3.3%)	1 (5%)	
2	0 (0%)	0 (0%)	
Stool smell			0.783
0	23 (76.7%)	16 (80%)	
1	7 (23.3%)	4 (20%)	
2	0 (0%)	0 (0%)	
Flatulence			0.243
0	28 (93.3%)	20 (100%)	
1	2 (6.7%)	0 (0%)	
2	0 (0%)	0 (0%)	
Abdominal pain			1.000
0	30 (100%)	20 (100%)	
1	0 (0%)	0 (0%)	
2	0 (0%)	0 (0%)	
Totally scores			0.164
0	15	15	
1	12	3	
2	1	2	
3	2	0	

Note: n.s. means not statically significant. *p < 0.05 vs NT group.

### Fecal levels of SCFAs in the ASD group distinctly vary from those in the NT group

**Figure 1:**
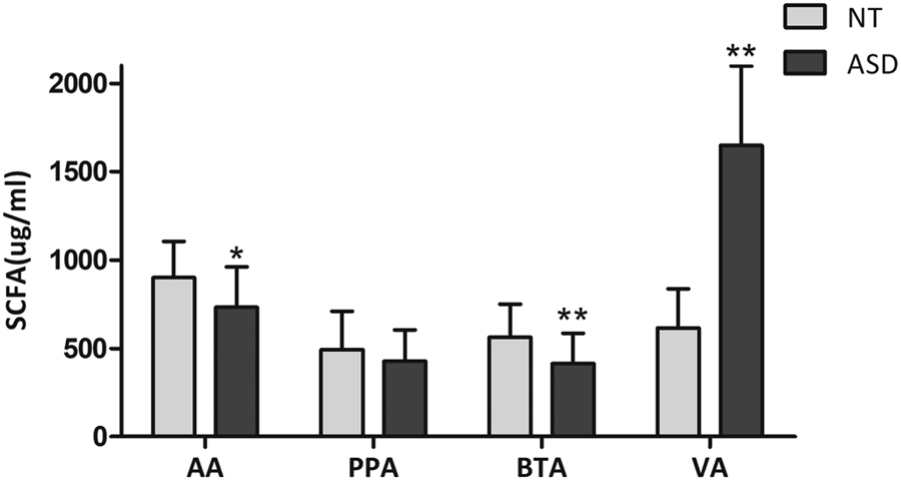
Fecal levels of short-chain fatty acids in participants. AA: acetic acid, PPA: propionic acid, BTA: butyrate, VA: valeric acid. Values are expressed as the mean ± SD, *p < 0.05 vs NT group; **p < 0.01 vs NT group.

The concentrations of acetic acid, propionic acid, butyric acid and valeric acid in feces are shown in Fig. 1. The acetic acid level was significantly decreased in autistic subjects (732.4 ± 229.8) compared with that in neurotypical controls (899.9 ± 204.4, *P* = 0.011). No significant differences were found in the levels of propionic acid between the two groups (*P* = 0.243). Compared with neurotypical children (563.3 ± 186.7), the butyrate concentration was significantly decreased in autistic children (413.2 ± 172.3, *P* = 0.005). However, the level of valeric acid in autistic children (1,646.9 ± 451.3) was markedly higher than those in neurotypical participants (615.8 ± 221.0, *P* < 0.001).

### Autistic subjects harbor an altered composition of the gut microbiota

**Figure 2:**
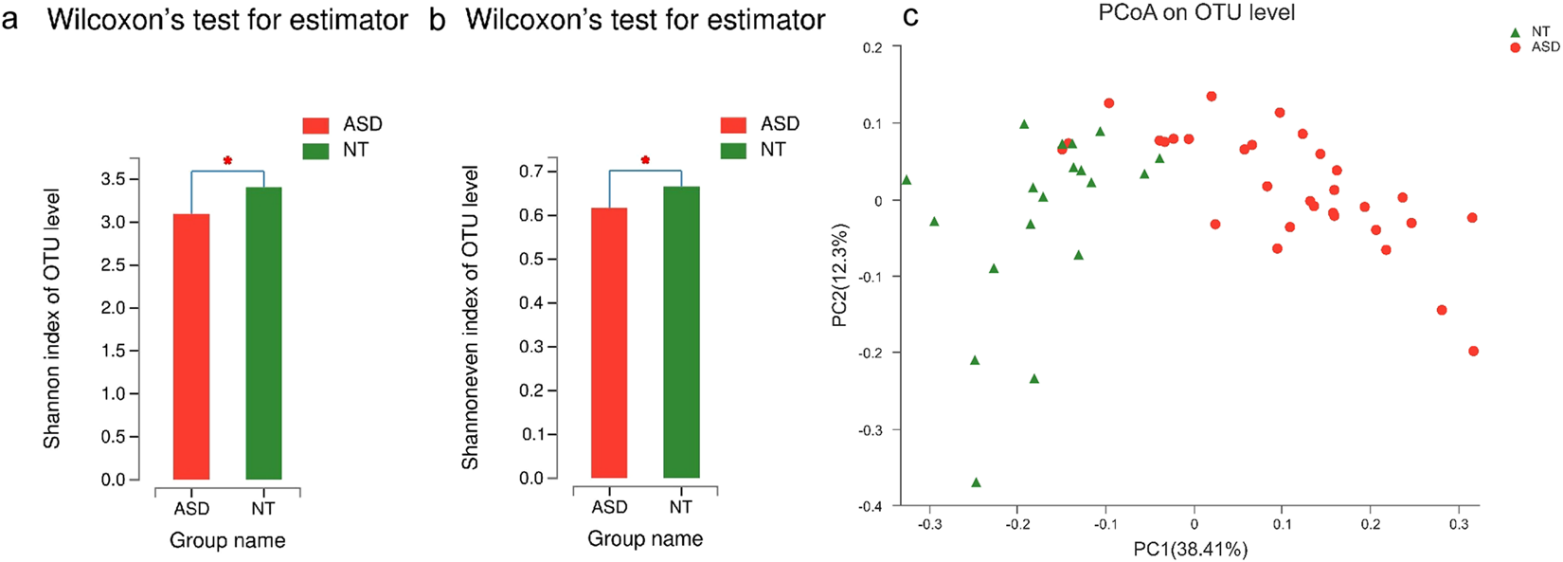
Gut microbiota diversity in ASD and NT subjects. (**a**) *Alpha* diversity based on the Shannon index of the OTU level (**b**) *Alpha* diversity based on the Shannoneven index of the OTU level. (**c**) PCoA of *beta* diversity based on the weighted UniFrac analysis of the OTU level. Autistic and neurotypical subjects are colored in red and green, respectively. *p < 0.05 vs NT group.

We obtained 1,922,441 sequences and 838,859,484 bases from 50 samples with an average of 38,449 sequences per sample. The analysis of *alpha* diversity revealed no significant differences using SOBS, Chao and ACE (reflecting species richness). However, the scores of the Shannon (Fig. 2a) and Shannoneven (Fig. 2b) indexes regarding the OTU levels in autistic samples decreased compared to those in neurotypical controls, suggesting that autistic children had less species diversity and evenness. Moreover, we performed principal coordinated analysis of *beta* diversity of the weighted UniFrac distance on the OTU data (Fig. 2c). The results revealed that the overall composition of the ASD gut microbiota was different from that of the NT gut microbiota.

**Figure 3:**
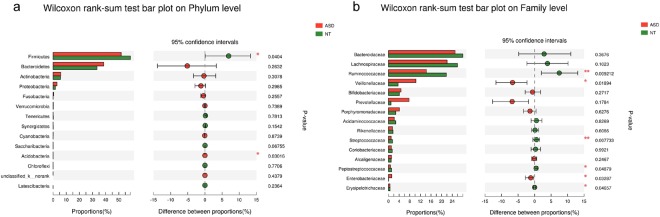
Abundances of taxa in ASD and NT participants. The mean relative abundances of taxa at the phylum (**a**) and family (**b**) levels in ASD and NT participants. Red and green bars indicate the mean relative abundances of taxa in autistic and neurotypical subjects, respectively. *p < 0.05 vs NT group; **p < 0.01 vs NT group.

We assessed the mean relative abundances of the taxa in ASD and NT to discriminant the specific alterations of the microbiota. At the phylum level, *Firmicutes* were significantly decreased in ASD (51.91%) compared to those in NT (58.82%), whereas *Acidobacteria* were considerably increased in ASD (0.01143%) compared to those in NT (0.00351%) (Fig. 3a). At the family level, there were no significant differences regarding the abundance of *Bacteroidaceae*, the most abundant family in the two groups. Furthermore, we observed six bacterial taxa that displayed different abundances between the two groups. *Veillonellaceae* and *Enterobacteriaceae* were enriched in autistic samples, whereas *Ruminococcaceae, Streptococcaceae, Peptostreptococcaceae* and *Erysipelotrichaceae* were significantly decreased in ASD with respect to those in NT (Fig. 3b).

**Figure 4:**
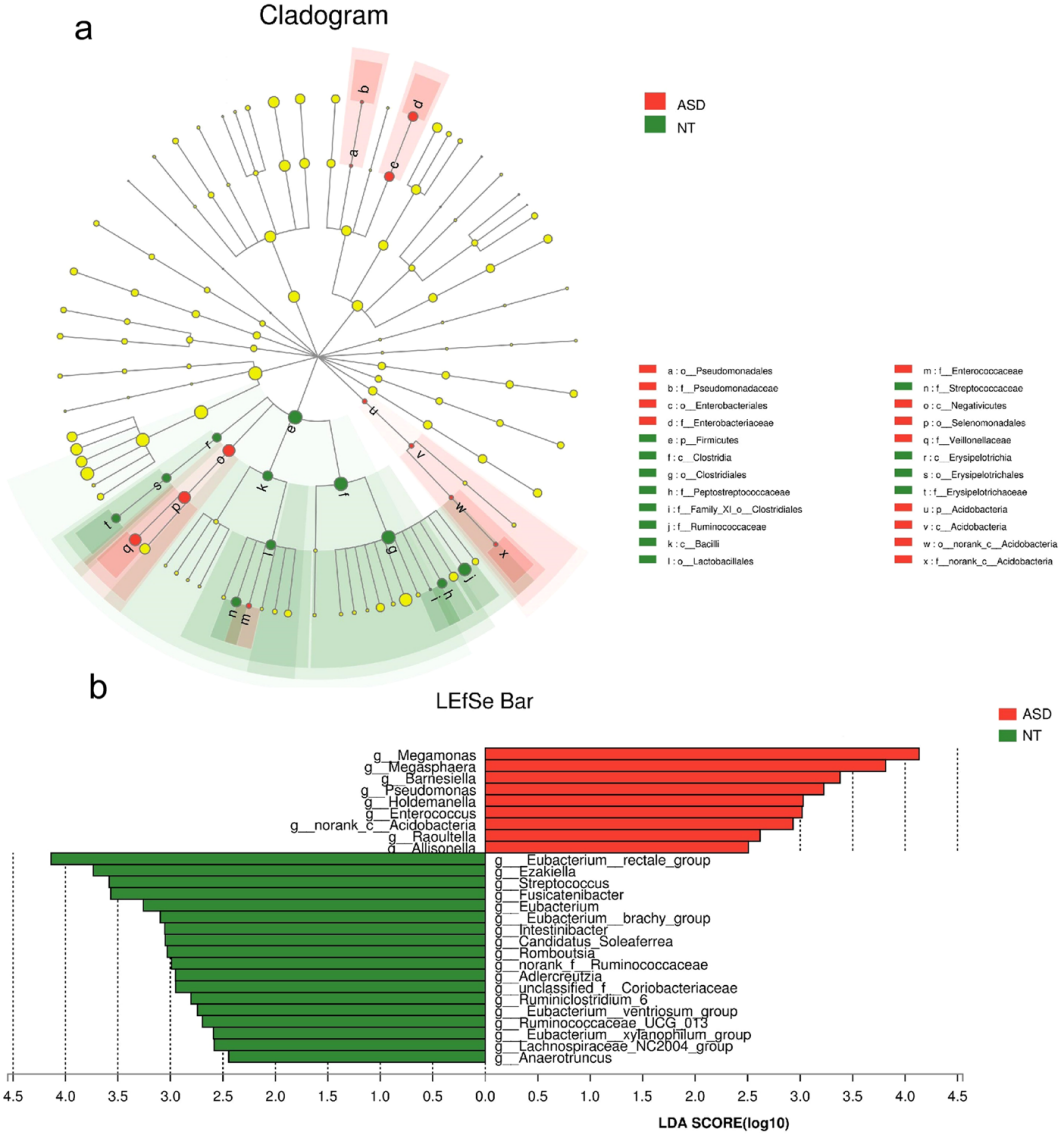
Differentially abundant bacterial taxa associated with the ASD and NT groups according to LEfSe analysis. The Cladogram generated by the LEfSe (from phylum to family level) and LDA scores (genus level) identify differentially abundant bacterial taxa associated with ASD and NT subjects. (**a**) Red and green dots indicate the bacterial taxa enriched in ASD and NT subjects, respectively. (**b**) Enriched bacterial taxa in ASD have positive LDA scores (red), and NT enriched bacterial taxa have negative scores (green). Only the taxa having an LDA > 2.0 are shown in the figure.

LEfSe was used to detect significant alterations in the bacterial composition associated with ASD and NT^16^. ASD subjects showed a significant increase in *Acidobacteria*, *Enterobacteriaceae* and *Pseudomonadaceae* apart from *Firmicutes*. The results also revealed a specific association between *Ruminococcaceae* and *Peptostreptococcaceae* within the order of *Clostridiales* in NT subjects. Regarding the order of *Lactobacillales*, *Enterococcaceae* and *Streptococcaceae* were overrepresented in ASD and NT subjects, respectively. In addition, there were a significant increase of the taxa *Veillonellaceae* and a significant decrease of *Erysipelotrichaceae* in ASD subjects compared to those in NT subjects. Furthermore, *Megamonas* was enriched in ASD subjects, whereas *Eubacterium* and *Lachnospiraceae-NC2004-group* were enriched in NT subjects at the genus level (Fig. 4).

### Correlation of the gut microbiota with SCFAs

**Figure 5:**
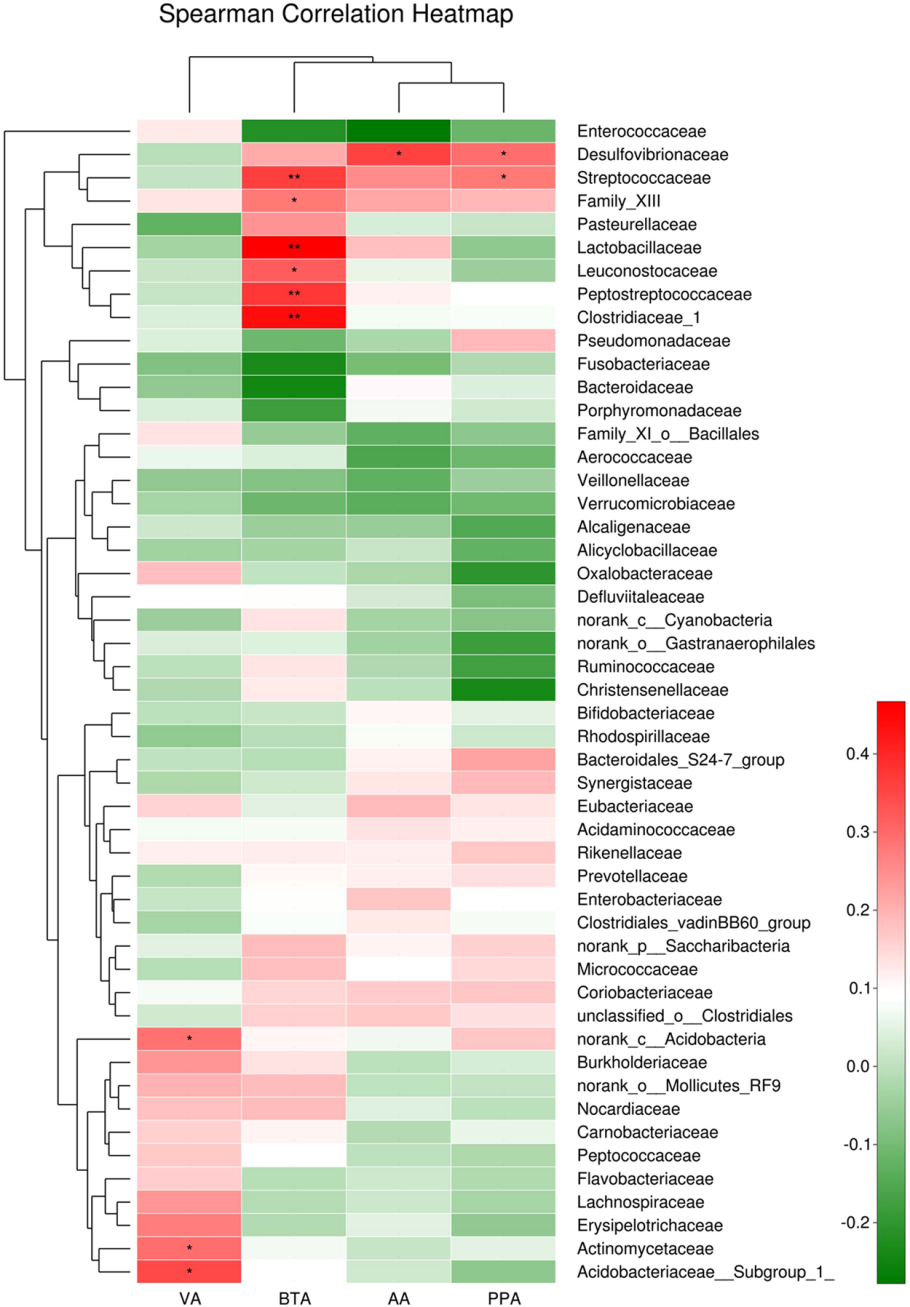
Correlation of the gut microbiota with the levels of fecal SCFAs. The heatmap shows the correlation coefficient between bacterial taxa and the level of fecal SCFAs at the family level. *p < 0.05; **p < 0.01.

Spearman correlation was used to associate the differentially abundant taxa with the fecal levels of SCFAs at the family level (shown in Fig. 5). *Acidobacteria* (significantly increased in ASD, rs = 0.349, *P* = 0.013) and *Actinomycetaceae* (rs = 0.298, *P* = 0.036) were positively correlated with valeric acid. *Streptococcaceae* (rs = 0.368, *P* = 0.009), *Peptostreptococcaceae* (rs = 0.378, *P* = 0.007), *Lactobacillaceae* (rs = 0.467, *P* = 0.001) and *Clostridiaceae_1* (rs = 0.441, *P* = 0.001), which were enriched in NT subjects, had a strong positive correlation with butyrate. In addition, we also observed a positive correlation between *Family_XIII* (rs = 0.281, *P* = 0.048), *Leuconostocaceae* (rs = 0.321, *P* = 0.023) and butyrate. *Desulfovibrionaceae* (rs = 0.295, *P* = 0.038) and *Streptococcaceae* (rs = 0.281, *P* = 0.048) were highly correlated with propionic acid. Moreover, *Desulfovibrionaceae* (rs = 0.36, *P* = 0.01) was also positively correlated with the level of acetic acid.

### Constipation alters the composition of the gut microbiota in ASD

**Figure 6:**
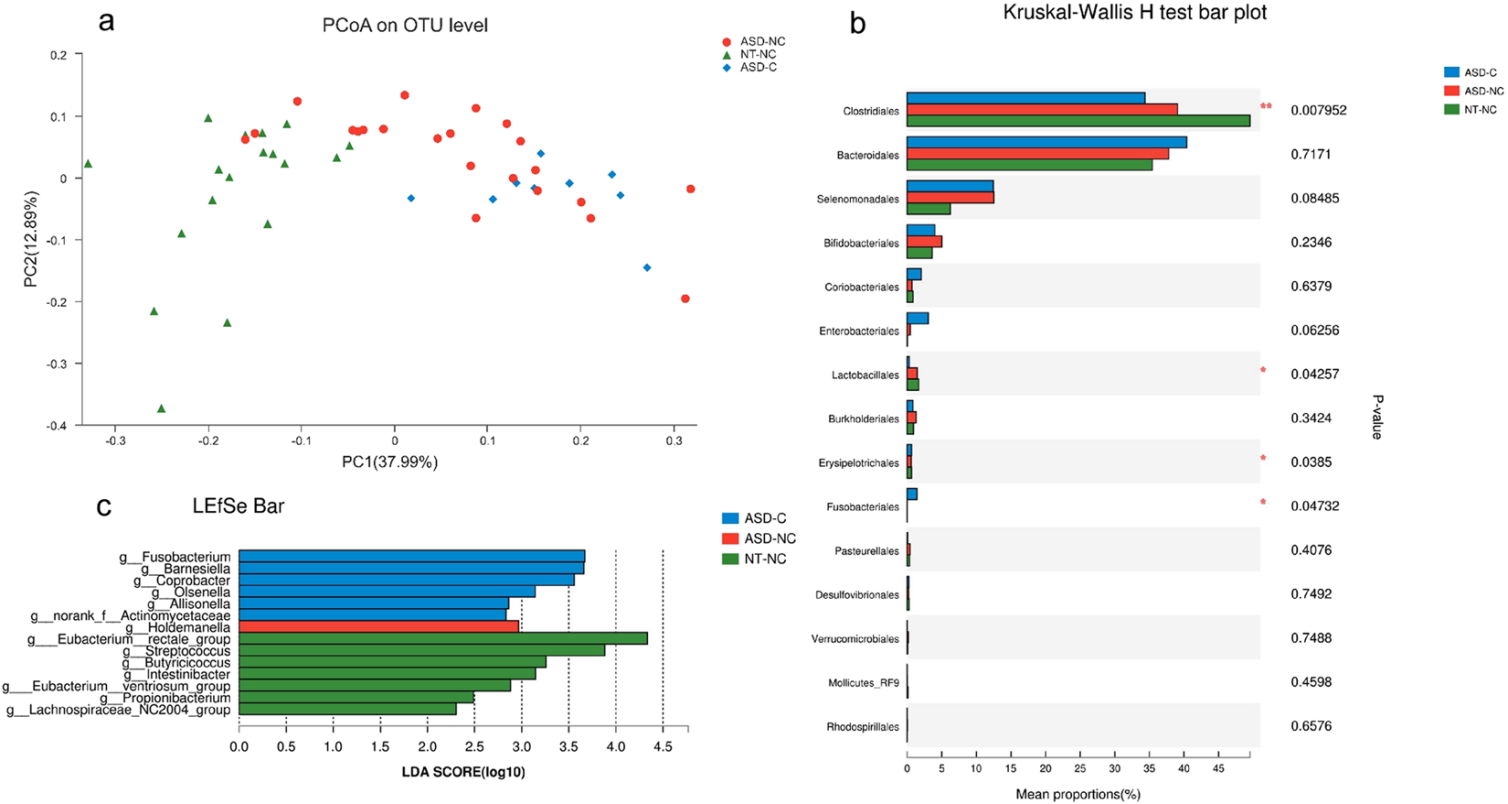
Constipation alters the composition of the gut microbiota in ASD. (**a**) PCoA of *beta* diversity based on the weighted UniFrac analysis on the OTU level among ASD-C, ASD-NC and NT-NC subjects. (**b**) The mean relative abundances of taxa at the order level in ASD-C, ASD-NC and NT-NC participants. (**c**) LDA scores indicate differentially abundant bacterial taxa associated with ASD-C, ASD-NC and NT-NC subjects (only the taxa having an LDA > 2.0 are shown in the figure). Blue, red and green bars indicate the bacterial taxa enriched in ASD-C, ASD-NC and NT-NC subjects, respectively. *p < 0.05 vs NT group; **p < 0.01 vs NT group.

Constipation was the significant GI symptom in ASD among the 6 GI symptoms evaluated in our study and is often reported as a common comorbidity in ASD. Considering the single constipated NT sample, which showed no statistical significance, we further compared the composition of the gut microbiota among constipated (ASD-C), non-constipated autistic (ASD-NC) and non-constipated neurotypical subjects (NT-NC). The species richness and diversity computed by analysis of *alpha* diversity did not show any significant differences among these three groups. When using the weighted UniFrac distances to calculate *beta* diversity, it was revealed that the microbiota of ASD-C deviated from those of ASD-NC and NT-NC (Fig. 6a). Order level analysis showed that *Clostridiales*, *Lactobacillales*, and *Erysipelotrichales* were represented in NT subjects and *Fusobacteriales* was represented in ASD-C subjects (Fig. 6b). We further analyzed genera with differentially abundant gene expression in ASD-C, ASD-NC and NT-NC samples using LEfSe. The results revealed a specific association of *Fusobacterium, Barnesiella, Coprobacter, Olsenella, Allisonella* and *Actinomycetaceae* with ASD-C subjects. *Holdemanella* was overrepresented in ASD-NC subjects compared to that in ASD-C and NT-NC subjects. The NT-NC microbiome was characterized by a preponderance of *Eubacterium_rectale_group, Streptococcus, Butyricicoccus, Eubacterium_ventriosum_group, Propionibacterium* and *Lachnospiraceae-NC2004-group* (Fig. 6c).

## Discussion

Several studies have noted alterations in the composition of the gut microbiota in ASD patients compared with neurotypical individuals. In the present study, we found less species diversity and evenness in autistic children and the overall structure of the gut microbiota compared with NT children. The results are similar to the study conducted by Kang^17^. Several studies demonstrate a significant increase in Firmicutes and a lower abundance of Bacteroidetes, resulting in an increased *Firmicutes/Bacteroidetes* ratio in autism spectrum disorder. We observed a considerable reduction of *Firmicutes* and a small proportion of the *Clostridium* genus in autistic children, consistent with the study of Finegold^18^. More specifically, *Ruminococcaceae, Peptostreptococcaceae, Lactobacillales, Streptococcaceae, Eubacterium* and *Lachnospiraceae-NC2004-group* were reduced within the phylum of *Firmicutes* in autistic subjects. A positive correlation between these bacteria and the fecal concentration of butyrate was determined in our study. Butyrate is mainly produced by anaerobic microbes from the fermentation of host-indigestible carbohydrates via the acetyl-coenzyme A (AcCoA) pathway^19^. Most butyrate-producing bacteria belong to *Clostridium* clusters IV and XIVa, including *Ruminococcaceae, Lachnospiraceae, Butyricicoccus* and *Eubacterium_rectale_group*^20,21^. Other *Clostridium* clusters also produce butyrate, including clusters I and XVI. However, these clusters produce lower levels of butyrate in comparison with *Clostridium* clusters IV and XIVa^22,23^. Moreover, Hakalehto reports that *Lactobacilli* contributes to the growth of butyrate-producing *clostridia*^24^. Apart from *Firmicutes*, we found that *Erysipelotrichaceae* was highly decreased in ASD, and Estaki indicated that *Erysipelotrichaceae* is also a key butyrate-produce member^25^.

Our data also showed a decrease in the fecal butyrate levels in ASD subjects. Butyrate is one of the major metabolites of the gut microbiome and regulates the activities of the microbiome-gut-brain axis. Butyrate regulates the integrity of gut barrier functions. Peng found that butyrate regulated AMP-activated protein kinase to reorganize and upregulate the expression of tight junction-associated protein^26^. Huang reported that butyrate, acting as a histone deacetylase-1 (HDAC-1) inhibitor, restored the intestinal epithelial barrier function by regulating the TWIK-related potassium channel-1 (Trek-1)^27^. Butyrate can strengthen mucosal immunity by its anti-inflammatory potential. In addition, a previous study indicates that butyrate restores BBB permeability by increasing histone acetylation and the expression of tight junction proteins^28^. Moreover, Kartsman discovered that the treatment with butyrate could regulate social behavior in an autism mouse model. Together, our results and the information mentioned above demonstrate that butyrate can regulate the activities of the gut-brain axis in ASD via specific transporters/receptors, regulating immune system and as an HDAC inhibitor.

We also observed increases in the abundance of taxa in the autistic group, including *Acidobacteria*, *Enterobacteriaceae*, *Pseudomonadaceae*, *Veillonellaceae* and *Megamonas*. *Acidobacteria* was positively correlated with valeric acid, leading to an increase in the fecal levels of valeric acid. Consistent with our research, a higher level of valeric acid has also been reported in ASD children^29^. It seems that valeric acid is another critical factor in autism, although its physiological functions are less reported regard to regulating the activities of the microbiome-gut-brain axis. Further studies on this point are needed. *Enterobacteriaceae* and *Pseudomonadaceae* are facultative anaerobic or aerobic fermentative Gram-negative bacilli. Most of these bacilli are opportunistic pathogens. *Enterobacteriaceae* overgrow in most inflammation models^30^. *Enterobacteriaceae* or their components (lipopolysaccharides) induce inflammation, aggravate intestinal injury and increase intestinal permeability, hypersensitivity, constipation and IBS^31^. Translocation of lipopolysaccharides into the blood can cause neuroinflammation and increase the BBB permeability. Lipopolysaccharides are associated with neurological disorders, such as major depression and Parkinson’s disease^32^. De Angelis also revealed an elevated abundance of *Enterobacteriaceae* in autistic children^33^.

The results of previous studies on fecal SCFAs are inconsistent in autism. Lower concentrations of SCFAs were reported in children with ASD by Adams^14^. In contrast to their study, Wang reported elevated SCFAs in autistic children^29^. This inconsistency might be due to the specific alteration of the gut microbiota in different populations and the technical or samples preparation differences in the different studies. Our results showed lower concentrations of fecal AA and BTA and a higher concentration of fecal VA in ASD individuals. The level of fecal SCFAs was mainly determined by the amount of SCFA producing bacteria and complex carbohydrates in the diet. In our study, all subjects had a normal diet. The lower levels of fecal BTA and higher levels of fecal VA are probably due to the variance of BTA-producing and VA-producing bacteria. We also found lower levels of fecal AA and a positive correlation between *Desulfovibrionaceae* and AA in ASD. Acetate is produced by various types of bacteria, including *Bacteroides*, *Bifidobacterium* and *Prevotella*^34^. Acetic acid promotes cholesterol synthesis in the liver. PPA is mainly produced by *Clostridia*, *Desulfovibrio*, *Propionibacterium* and *Bacteroides*^35^. We found that *Desulfovibrionaceae* and *Streptococcaceae* were positively correlated with PPA. PPA can induce ASD-like behaviors through intracerebroventricular and intraventricular administration^36,37^. PPA induces impaired social behavior through regulating neurotransmitters, such as dopamine^38^. We did not find a significant alteration of fecal PPA in autistic subjects in our study, probably due to ethnic and dietary structure difference in Chinese children compared to others.

Despite the report of constipation, diarrhea, abdominal pain, flatulence and other GI disorders in ASD patients by other researchers, we only observed that constipation was significantly increased in the ASD group compared to that in the control group. Holingue indicates that constipation is the most prevalent GI symptom in ASD^39^. We found enriched *Fusobacterium*, *Barnesiella, Coprobacter* and valeric acid-associated bacteria (*Actinomycetaceae*) and reduced butyrate-producing taxa in ASD-C subjects, suggesting that constipation has a notable effect on the gut microbial composition within ASD subjects.

In summary, constipation is the GI comorbidity in ASD children in our study. We observed a shifted gut microbiota and its association with fecal SCFAs in ASD individuals. Therapeutic strategies that modulate the gut microbiota and SCFAs, especially butyrate-producing bacteria, may have potential to relieve ASD and GI-related symptoms in ASD.

## Materials and Methods

### Study participants

We recruited 30 (2.5–18 years old) autistic subjects (ASD) from the Fifth and Third Affiliated Hospital of Zhengzhou University. Twenty age- and sex-matched healthy volunteers were accepted as neurotypical controls (NT). The diagnosis of autism was established according to DSM-V (Diagnostic and Statistical Manual of Mental Disorders, 5^th^ Edition)^1^ and ICD-10 (International Statistics Classification of Diseases and Related Health Problems, 10^th^ Revision)^40^ by two experienced child neuropsychiatrists. Neurotypical controls were typically developing children, without an autism diagnosis and not directly related to an autistic individual. The exclusion criteria included a history of nutritional supplements and special diets, presence of significant physical abnormalities, and neurological disorders of known etiology. Participants in this study were not treated with antibiotics, antifungals, probiotics or prebiotics for at least three months before sampling. All methods were carried out in accordance with the relevant guidelines and regulations. All experimental protocols were approved by the Ethical Committee of the Fifth Affiliated Hospital of Zhengzhou University (No. 2016-1001). Informed written constent was obtained from the parents and/or legal guardians of the enrolled participants.

### Severity scales of GI symptoms

Gastrointestinal symptoms were assessed following a modified version of the 6-GI Severity Index^14^, including constipation, diarrhea, average stool consistency, stool smell, flatulence and abdominal pain.

### Fecal sample collection

Fecal samples were collected and transported to our laboratory for processing within 30 minutes. The 200 mg stool was preserved in fecal bacteria DNA storage tubes (Tinygene Biological Company, China) and stored at −80 °C for 16S rRNA sequencing. The 600 mg stool was preserved in sterile tubes and immediately frozen at −80 °C for further SCFA analysis.

### Fecal SCFA analysis

The short-chain fatty acids were extracted as described previously^41^. Briefly, 300 mg stool was homogenized with 1 ml ddH_2_O and centrifuged at 12,000 g for 10 min. The supernatant was homogenized with 100 μl concentrated HCl and subsequently extracted for 20 min using 5 ml of diethyl ether. After centrifugation (3,500 rpm, 10 min), we mixed the organic phase with 500 μl NaOH (1 M). Then, the organic phase was extracted and centrifuged again. The aqueous phase was mixed with 100 μl concentrated HCl and filtered through a 0.22 μm filter. A high-performance liquid chromatography system (HPLC, Waters Alliance e2695, Milford, USA) with a UV detector (Waters 2468, Milford, USA) was used with a Venusil ASB C_18_ (4.6 × 250 mm, 5 μm, China) column at a flow rate of 1 ml/min at 210 nm and 30 °C. The mobile phase consisted of 0.01% H_3_PO4 in HPLC-grade water (A) and methanol (B). All samples were evaluated in duplicate.

### DNA extraction and amplification of the V3-V4 region of the bacterial 16S rRNA gene

Microbial DNA was extracted from 200 mg fecal samples using the QIAamp Fast DNA Stool Mini Kit (QIAamp, California, USA). The final DNA concentration and purity were detected by a NanoDrop 2000 UV (Thermo Scientific, Wilmington, USA). We amplified the bacterial 16S rRNA gene using primers specific for the V3-V4 hypervariable regions (338 F: 5′-ACTCCTACGGGAGGCAGCAG-3′ and 806 R: 5′-GGACTACHVGGGTWTCTAAT-3′) in a thermocycler PCR system (GeneAmp 9700, ABI, USA). The amplicons were extracted and further purified using the AxyPrep DNA Gel Extraction Kit (Axygen Biosciences, Union City, CA, USA) and quantified with a QuantiFluor™-ST (Promega, USA) following the manufacturer’s instructions.

### Illumina MiSeq sequencing

The sequencing data were pooled equimolarly and paired-end sequenced (2 × 300) on an Illumina MiSeq platform (Illumina, San Diego, USA) according to the standard protocols by Majorbio Bio-Pharm Technology Co. Ltd. (Shanghai, China). The raw reads were deposited in the NCBI Sequence Read Archive (SRA) database. Raw fastq files were demultiplexed and quality-filtered by Trimmomatic. The reads were merged by FLASH as described previously^42^. Operational taxonomic units (OTUs) were clustered with a 97% similarity cutoff using UPARSE (version 7.1 http://drive5.com/uparse/), and chimeric sequences were identified and removed using UCHIME. The taxonomy of each 16S rRNA gene sequence was analyzed by the RDP Classifier algorithm (http://rdp.cme.msu.edu/) against the Silva (SSU123) 16S rRNA database using a confidence threshold of 70%^43^.

### Bioinformatic analysis

All sequencing data were computed using the R package (V.2.15.3). Rarefaction was applied to the OTUs to reduce sampling heterogeneity for further alpha and beta diversity calculations. Principal coordinates analysis (PCoA) was used to assess species composition dissimilarity by the weighted UniFrac. The linear discriminant analysis effect size (LEfSe), an algorithm for high-dimensional biomarker discovery, was computed to identify differentially abundant taxa between two groups. The linear discriminant analysis (LDA) effect size (LEfSe) method was used to estimate the effect of each differentially-abundant taxon and discriminate the most biological one^16^. The Wilcoxon rank-sum test (for two groups) or Kruskal-Wallis test (for more than two groups) was used for comparisons. Additionally, Spearman correlation was used to associate abundant differential taxa with SCFAs.

### Statistical analysis

Other data were analyzed using SPSS20.0 software (IBM Corp., Armonk, N.Y., USA). Comparison of data between autistic and neurotypical subjects was conducted using Student’s *t*-test (qualitative data, equal variance), Welch’s *t*-test (qualitative data, unknown variance) or the Chi-square test (qualitative data). Data are presented as the mean ± SD. Significance was set at 0.05.
